# A clinical study of non-bioartificial liver DPMAES support system in hepatitis B-related acute-on-chronic liver failure

**DOI:** 10.1038/s41598-024-52206-0

**Published:** 2024-01-20

**Authors:** Xianwen Cheng, Yanrong Zhan, YaoShun Liu, Xia Zeng, Zhendong Wang, Feng Wang, Ya Mao, Song Na

**Affiliations:** 1 Ankang Hospital of Traditional Chinese Medicine, Ankang, 725000 Shaanxi China; 2 Shaanxi University of Chinese Medicine, Xianyang, 712000 Shaanxi China

**Keywords:** Diseases, Gastroenterology, Hepatitis, Liver, Liver diseases

## Abstract

This study aims to observe the clinical efficacy of the dual plasma molecular adsorption exchange system (DPMAES) in patients with hepatitis B virus-related acute-on-chronic liver failure (HBV-ACLF), with a focus on its regulatory effect on cytokine storm. A total of 60 HBV-ACLF patients were enrolled in this study. The observation group, comprising 30 patients, received DPMAES treatment, while the control group underwent PE treatment. We compared the efficacy changes between the two groups post-treatment. A total of 55 HBV-ACLF patients who completed the study were analyzed, Patients treated with DPMAES showed significant improvements in clinical outcomes. After DPMAES treatment, HBV-ACLF patients exhibited notably 90 day survival rate increased by 18% compared to those in the PE group. Moreover, total bilirubin levels decreased markedly, albumin and platelet levels increased compared to the PE group. After DPMAES treatment, the patient showed a significant decrease in inflammatory cytokine IL-6 (t = 5.046, *P* < 0.001) and a significant decrease in procalcitonin (t = 4.66, *P* < 0.001). DPMAES was more effective than PE in rapidly reducing TBiL, improving coagulation function and mitigating cytokine storm. It maintained platelet stability more effectively while minimizing albumin consumption to a greater extent, significantly improved 90-day survival.

*Trial registration*: Chinese Clinical Trial Registry, ChiCTR2300076117.

## Introduction

Liver failure is a severe condition marked by significantly impaired synthesis, detoxification, excretion, and biotransformation functions. It manifests primarily as coagulation disorders, jaundice, hepatic encephalopathy, and hepatorenal syndrome, with mortalities reaching 60–70%^1^. Acute-on-chronic liver failure (ACLF) is an acute decompensation of liver function in patients with chronic liver disease caused by infection, bleeding, and non-standard antiviral therapy. In China, hepatitis B virus (HBV) is the main cause of liver failure^2^, affecting about 230,000 people annually. Currently, there is a lack of specific drugs for medical treatment. Liver transplantation is an effective treatment method; however, the limited availability of donor organs and the capabilities of transplantation teams contribute to its relatively restricted global clinical coverage, resulting in only a fraction of patients benefiting from this procedure. Non-biological artificial liver is an effective and widely employed clinical treatment method for liver failure. ACLF manifests varying degrees of systemic inflammatory response syndrome (SIRS), gut-derived endotoxins (GN), immune damage, and ischemic hypoxic damage. With the exacerbation of SIRS, GN, cytokine storm (CS), and other injuries, both local and systemic circulatory disorders can occur in the body, leading to cytokine storm sepsis (CSS) injury mechanisms. CSS differs from traditional sepsis, which is defined as a systemic inflammatory response accompanied by organ dysfunction caused by infection. Currently, the mechanism of CSS has not been fully elucidated in the field of ACLF research, and CSS is closely related to the inflammatory response mediated by liver sinusoidal macrophages. Dual plasma molecular adsorption exchange system (DPMAS), a novel non-biological artificial liver technology, stands out as one of the effective management methods. In 2018, it was included in the Chinese Guideline for Diagnosis and Treatment of Liver Failure^3^. DPMAS sequential or combined plasma exchange (PE), although effective, has drawbacks such as high costs, complex operations, time-consuming, and high risk of infection. Our team innovatively developed a single system DPMAES, overcoming these limitations by integrating full-dose plasma exchange within the same equipment. This patented technology (ZL202122215714.2) has been clinically proven over three years to deliver significant clinical efficacy. The DPMAES mode of artificial liver possesses a dual effect of plasma purification and plasma exchange, which can accomplish adsorption, filtration, and exchange simultaneously, and plays an important regulatory role in the mechanism of CSS damage in liver failure. Therefore, the present study aims to explore the clinical efficacy of DPMAES in ACLF, thereby determining the regulatory effect of DPMAES on CSS and its significance for the liver.

## Patients and methods

### General information

The trial was registered in the WHO International Clinical Trial Registry Platform with registration number ChiCTR2300076117 (date of registration: 25/09/2023). Informed consent was obtained from the patients before treatment. The study followed the guidelines of Good Clinical Practices in China and received approval from the Human Biomedical Research Ethics Branch of Ankang Traditional Chinese Medicine Hospital (2021AKZYLL-048-01). This randomized controlled trial prospectively included 60 hospitalized patients from the Department of Gastroenterology at Ankang Hospital of Traditional Chinese Medicine between March 2019 and June 2022. The patients were randomly divided into an observation group (n = 30) and a control group (n = 30) using a random number table.

### Diagnostic, inclusion, and exclusion criteria

The inclusion criteria were formulated based on the Guideline for Diagnosis and Treatment of Liver Failure formulated by the Chinese Society of Infectious Diseases of Chinese Medical Association in 2018 and the Acute-on-Chronic Liver Failure Clinical Guidelines in the United States^4^, in which liver failure is categorized to early-stage, pre-stage, mid-stage, and late-stage. Acute-on-chronic liver failure (ACLF) is defined as a syndrome characterized by acute worsening of jaundice and coagulation dysfunction on the basis of chronic liver disease, accompanied by additional complications hepatic encephalopathy, ascites, electrolyte imbalance, and infections, as well as extrahepatic organ dysfunction. The jaundice rapidly intensifies, with serum total bilirubin (TBil) levels reaching ≥ 10 times the upper limit of normal (ULN) or increasing by ≥ 17.1 μmol/L per day. Additionally, there are signs of bleeding, with prothrombin activity (PTA) ≤ 40% (or international normalized ratio [INR] ≥ 1.5). Inclusion criteria were as follows: pre-stage, early-stage, and mid-stage of ACLF caused by hepatitis B virus infection. Exclusion criteria were as follows: co-infection with human immunodeficiency virus (HIV) or other viral infections, malignant tumors, acute attacks of cardiovascular and cerebrovascular diseases, pregnancy or lactation, mental disorders, end-stage liver disease, severe active bleeding or disseminated intravascular coagulation, hypersensitivity to blood products or medications used during treatment (such as plasma, heparin, and fish protein), circulatory disorders, recent stroke with instability.

### Excluded and dropout cases

Patients were excluded if they failed to adhere to the treatment regimen or complete regular follow-up (resulting in missing data that could affect efficacy and safety assessments), were lost to follow-up during the observation period, consumed f medications or substances (e.g., alcohol) that could affect outcome, experienced severe adverse events requiring study withdrawal, or voluntarily withdrew from the study.

### Research methods

The observation group was treated with DPMAES while the control group received PE therapy. All patients received routine comprehensive internal medicine treatments, including bed rest, nutritional support, hepatoprotective medications, and symptomatic treatment. Antiviral therapy with nucleoside analogs (e.g., Tenofovir Alafenamide Fumarate Tablets 25 mg, H20180060) was also administered. For DPMAES treatment, the artificial liver treatment machine JUN-55X from Japan was used. The initial treatment involved the placement of a femoral vein single-needle double-lumen catheter (Able 11.5 cm × 13.5 cm). (1) DPMAES mode: After plasma separation using the OP-08 primary plasma separator from Asahi Kasei, bilirubin and inflammatory mediators were adsorbed using BS330 bilirubin adsorption column (Zhuhai Livzon Medical Bio-material Co. Ltd.) and HA330-II resin hemoperfusion cartridge (Zhuhai Livzon Medical Bio-material Co. Ltd.). During the initial DPMAS treatment, the PA mode was selected. The exchange reservoir end of the "Y"-shaped tubing was clamped, while the closed end of the three-way valve on the extension tube was directed towards the waste bag and the open end connected to the venous reservoir. The treatment lasted 3–4 h during which approximately 5 L of plasma adsorbed. For subsequent sequential PE treatment, the PE mode was chosen. External pressure measurement was disabled. The exchange reservoir of the "Y"-shaped tubing was opened, and the outlet of the three-way valve was directed towards the waste bag while the closed end was towards the venous reservoir. Virus-inactivated frozen plasma (2500–3000 mL) of the same blood type was used for plasma separation, adsorption, filtration, and extensive exchange in a single pass. (2) PE: After plasma separation using the OP-08 primary plasma separator from Asahi Kasei, the blood flow rate was set at 100–120 mL/min, and the plasma separation rate was 20–30 mL/min. Plasma exchange was performed using virus-inactivated frozen plasma (2500–3000 mL) of the same blood type and 10g of human albumin. The treatment duration for PE was approximately 3 h. (3) To prevent plasma allergy, 5 mg of dexamethasone (Jin H20130301, 5 mg, Pfizer Belgium) and 5 mL of 5% calcium gluconate were administered intravenously before treatment. The dose of heparin for anticoagulation was adjusted based on coagulation function throughout the procedure. During PE, if PTA was < 20%, no heparin was used. For PTA between 20 and 40%, an initial dose of 625–1250 U (5–10 mg) was given. For PTA between 40 and 80%, an initial dose of 1250–2500 U (10–20 mg) was followed by a maintenance dose of 312.5–625 U/h (2.5–5.0 mg/h). The DPMAES treatment could involve similar dosage adjustments based on factors such as platelet count and anticoagulant enzyme activity. Low-molecular-weight heparin sodium injection (H20140281, 4000U, Alfa Wassermann SpA, Italy) was used as the anticoagulant. Throughout the procedure, continuous monitoring of electrocardiography, blood pressure, and oxygen saturation was conducted to closely observe changes in patients' condition.

### Observed parameters

1. Primary endpoint: The 30-day and 90-day survival rates after artificial liver treatment were recorded (patients who were discharged due to deteriorating conditions were considered as disease-related deaths).
2. Secondary endpoints: Blood samples were collected before and after treatment (morning of the treatment day and next morning, respectively). Biochemical parameters such as TBil were analyzed using an automated biochemical analyzer with the corresponding reagent (code: E1006). INR was measured using an automated coagulation analyzer (CS5100, Sysmex, Japan). Levels of cytokines like IL-6 were measured using ELISA. Liver function, coagulation function, and blood routine tests were repeated on day 28 and day 90. HBVDNA levels were tested before treatment and at 7 days, 28 days, and 90 days after treatment.
3. Safety parameters included blood routine, renal function, vital signs, coagulation in tubing and filters, transmembrane pressure (TMP), bleeding events, infections, etc.

### Statistical analysis

All variables were expressed as mean ± standard deviation ($\overline{{\text{X}}} \pm {\text{S}}$). Paired t-tests were used for within-group comparisons against the baseline, while independent sample t-tests were used for between-group comparisons of differences before and after treatment. Survival rates were analyzed using Logistic regression analysis with calculation of relative risk (RR) values. Statistical significance was set at *p* < 0.05. Data were analyzed using SPSS 21.0 statistical software.

## Results

### Comparison of general characteristics between the two groups

A total of 55 patients with hepatitis B virus related acute-on-chronic liver failure (HBV-ACLF) completed the study, with similar distributions of gender, age, and disease duration between the observation (n = 28) and control groups (n = 27) (*P* > 0.05). There were 32 males (17 in the observation group and 15 in the control group) and 23 females (11 in the observation group and 12 in the control group). The age ranged from 20 to 68 years, with mean ages of 36.50 ± 9.55 years in the observation group and 38.75 ± 10.26 years in the control group. The disease duration ranged from 1 to 8 years, with mean durations of 4.85 ± 1.92 years in the observation group and 6.28 ± 2.74 years in the control group.

### Comparison of survival rates between the two groups

Logistic regression analysis showed that compared to the control group, the 30-day survival rate in the observation group increased by 5%, but without statistical significance (*p* > 0.05). At 90 days, the observation group had a significantly higher survival rate, with an increase of 18% (*P* < 0.05). The results are shown in Table 1.

**Table 1:** Comparison of survival rates between the two groups.

Group	N	30-Day survival rate (%) n	90-Day survival rate (%) n
Observation group	28	85.71% (24/28)	78.57% (22/28)△
Control group	27	81.48% (22/27)	66.67% (18/27)
RR value (95%CI)		1.05	1.18
*P* value		> 0.05	< 0.05

Compared with the control group in the same period, △*P* < 0.05.

### Comparison of liver function, coagulation, and platelet parameters between the two groups

TBil: Both groups experienced significant declines in TBil after treatment (*P < 0.05), with a greater decrease in the observation group (△*P* < 0.01). INR: Significant reductions in INR were observed before and after treatment within both groups, but there were no statistically significant differences between the groups. ALB: The observation group showed a non-significant increase in ALB levels after treatment (see Table 2) while the control group exhibited a non-significant decrease. However, compared to the control group, the observation group exhibited a statistically significant increase in ALB after treatment (△*P* = 0.002). Platelet: Within-group comparisons showed a significant platelet recovery after treatment in the observation group, with statistical significance. However, no significant differences were observed between the observation and control groups or within the control group.

**Table 2:** Comparison of liver function, coagulation, and platelets between two groups.

Group		N	TBiL (umol/L)	INR	ALB (g/L)	PLT/(10^9^/L)
Observation group	Before treatment	28	291.31 ± 65.61	1.58 ± 0.53	29.72 ± 4.56	84.12 ± 23.58
	After treatment		131.15 ± 37.84*△	1.26 ± 0.37*	31.64 ± 3.95△	102.65 ± 29.84*
Control group	Before treatment	27	307.73 ± 73.25	1.63 ± 0.62	30.15 ± 3.94	87.56 ± 26.53
	After treatment		183.44 ± 35.58*	1.28 ± 0.32*	28.45 ± 3.73	96.62 ± 30.75
T value			5.274	0.21	3.21	0.4
*P* value			< 0.01	0.08	0.002	0.74
t_1_ value			11.19	3.44	1.68	2.58
*P*_*1*_ value			< 0.0001	0.001	0.09	0.01
t_2_ value			7.908	2.61	1.7	1.16
*P*_2_ value			< 0.0001	0.01	0.09	0.25

TBiL, total bilirubin; ALB, albumin; PLT, platelet. Paired t-test was used for comparison between groups in the same group, and independent samples t-test was used for comparison between two groups; t-value and p-value were meaningful comparisons between the two groups; t_1_ value and *P*_1_ value were compared with the observation group before treatment, The t_2_ value and *P*_2_ value were compared before treatment in the control group. Within-group comparison, *P < 0.05; between-group comparison, △*P* < 0.05.

### Comparison of inflammatory marker levels before and after treatment between the two groups

Before treatment, neither groups showed significant differences in IL-6 and procalcitonin (PCT) levels. After treatment, both groups showed significant decreases in IL-6 and PCT (*P* < 0.05). Compared to the control group, post-treatment levels of IL-6 (t = 5.046, *P* < 0.0001) and PCT (t = 4.66, *P* < 0.0001) showed a more pronounced decrease in the observation group than in the control group (see Table 3).

**Table 3:** Comparison of inflammatory factor levels before and after treatment in two groups of patients ($\overline{{\text{X}}} \pm {\text{S}}$).

Group		Sample	IL-6 (ng/ml)	PCT (ng/ml)
Observation group	Before treatment	28	0.217 ± 0.085	1.25 ± 0.67
	After treatment		0.095 ± 0.037*△	0.48 ± 0.23*△
Control group	Before treatment	27	0.205 ± 0.092	1.37 ± 0.75
	After treatment		0.157 ± 0.053*	0.85 ± 0.36*
T value			5.046	4.55
*P* value			< 0.0001	< 0.0001
t_1_ value			6.94	5.75
*P*_*1*_ value			< 0.0001	< 0.001
t_2_ value			2.34	3.25
*P*_*2*_ value			0.02	0.002

IL-6, interleukin-6; PCT, procalcitonin. Paired t-test was used for comparison between groups in the same group, and independent samples t-test was used for comparison between two groups; t-value and *p*-value were meaningful comparisons between the two groups; t_1_ value and *P*_1_ value were compared with the observation group before treatment, The t_2_ value and *P*_2_ value were compared before treatment in the control group. Within-group comparison, *P < 0.05; between-group comparison, △*P* < 0.05.

### Comparison of HBV-DNA levels (log10 IU/mL) between the two groups

Both groups experienced significantly lower HBV-DNA levels at 7, 30, and 90 days compared to baseline (*P* < 0.05). Notably, at 90 days, the observation group exhibited significantly lower viral load compared to the control group (*P* < 0.05, t = 7.3). The results are shown in Table 4.

**Table 4:** Comparison of high sensitivity HBV-DNA (log10 IU/ml) between two groups before and after treatment ($\overline{{\text{X}}} \pm {\text{S}}$).

Item	Time	Observation group	Control group
High sensitivity HBV-DNA	Before treatment	7.42 ± 1.87	7.14 ± 1.62
	Day 7	5.72 ± 1.35△	5.58 ± 1.69△
	Day 30	3.48 ± 1.36△	3.89 ± 1.54△
	Day 90	1.25 ± 0.45△*	2.27 ± 0.58△

Within-group comparison, *P < 0.05; between-group comparison, △*P* < 0.05.

### Comparison of adverse events and risk of death during treatment between the two groups

By the study end, 40 patients showed clinical improvement (see Table 5). In the observation group, 28 patients completed DPMAES treatment with a total of 94 treatment sessions, resulting in 12 adverse reactions (12.76%) including 8 cases of allergic reactions, 2 cases of blood pressure and 2 cases of vomiting. After administration of symptomatic treatments including glucose calcium supplementation, volume expansion, blood flow rate reduction, and antiemetics, general symptoms were resolved without affecting the treatment process. In the control group, 27 patients completed PE treatment with a total of 87 treatment sessions, resulting in 16 adverse reactions (18.39%), including 14 cases of allergic reactions and 2 cases of vomiting. The incidence of adverse events was higher in the control group, but the difference was not statistically significant (*P* > 0.05). The overall survival rate at day 90 was 72.72%. Among the 15 deceased patients: 80% (12/15) had alpha-fetoprotein (AFP) < 35 ng/mL, 46.67% (7/15) were classified as having C-type ACLF, 53.33% (8/15) had lactate > 3.4 mmol/L, and 93.33% (14/15) had complement C3 < 0.45 g/L.

**Table 5:** Univariate analysis of mortality risk comparison.

Group		N	AFP (ng/ml)	complement C3 (g/L)	Lac (mmol/L)	C-type ACLF (%)
Improvement group	Baseline	40	152.85 ± 65.28	0.63 ± 0.17*	1.56 ± 0.65	7.5%
	After treatment		179.54 ± 73.96△	0.85 ± 0.36△	1.48 ± 0.24△	7.5%
Death group	Baseline	15	39.26 ± 8.56	0.51 ± 0.12	2.84 ± 1.03	46.67%
	After treatment		34.73 ± 3.71	0.45 ± 0.07	4.63 ± 1.85*	46.67%△
T value			7.53	4.29	10.94	RR = 6.2
*P* value			0.0000	0.000	0.0000	< 0.05
t_1_ value			1.71	3.49	0.73	
*P*_*1*_ value			0.09	0.0008	0.46	
t_2_ value			1.88	1.8	3.27	
*P*_*2*_ value			0.07	0.07	0.003	

AFP, alpha-fetoprotein; Lac, lactate; ACLE, acute-on-chronic liver failure. Paired t-test was used for comparison between groups in the same group, and independent samples t-test was used for comparison between two groups; t-value and *p*-value were meaningful comparisons between the two groups; t_1_ value and *P*_1_ value were compared with the observation group before treatment, The t_2_ value and *P*_2_ value were compared before treatment in the control group. Within-group comparison, *P < 0.05; between-group comparison, △*P* < 0.05.

## Discussion

ACLF, the most prevalent and deadliest severe liver disease in China, lacks effective specific pharmacological treatments. Early detection, diagnosis, and treatment are crucial, emphasizing etiological treatment, comprehensive support, prevention and management of complications, non-biological artificial liver support and liver transplantation^5,6^. Non-biological artificial liver plays a crucial role in the entire treatment process, and even liver transplantation, without the adjunctive support of artificial liver, would ultimately yield lesser benefits. A study conducted by Zhejiang University in China reviewed data from 166 patients with chronic liver failure and ACLF over the past 8 years, and the results showed that a non-biological artificial liver support system combined with transplantation significantly improved short-term survival rates. One partial mechanism is that artificial liver treatment reduces systemic inflammation and improves the internal environment^7^. While the combination of DPMAES with PE has shown promise, it often yields unsatisfactory results in patients with C-type ACLF, PT activity less than 20%, and TBiL levels exceeding 300 umol/L. Most these patients require 3–4 treatment sessions to achieve better clinical outcomes, which could involve potentially high costs^8^. To address these limitations, our team has innovatively developed the DPMAES system which was patented in 2021, enabling simultaneous dual molecular adsorption and extensive plasma exchange. This not only reduces treatment costs by approximately ¥3000 per session but also exhibits promising clinical efficacy, as will be discussed in detail in the following.

The severity of HBV-ACLF closely correlates with viral load. Early research led by Academician Li Lanjuan^9,10^ suggests that PE therapy is more effective in reducing viral loads compared with monotherapy using antivirals. Both PE and DPMAES have been shown to reduce serum HBV load. In our study, we observed a significant reduction in HBV-DNA levels in both patient groups after seven days of treatment. This decline is likely due to the antiviral medication and plasma exchange therapy used for viral removal. This viral clearance is crucial, but maintaining a favorable internal environment is equally important for hepatocyte regeneration. The disturbance of the internal environment is primarily attributed to immune-metabolic disorders in liver failure, which can cause local and systemic inflammation of the liver and its microcirculation disorders, subsequently inducing and exacerbating CCS. In the study of acetaminophen-induced ACLF, it is suggested that immune inflammation, metabolic disorder, bile stasis, and microcirculation disorders are the principal factors exacerbating liver failure. Treatment strategies targeting immune regulation, metabolic balance, vascular remodeling, and bile duct repair have the potential to lower the high mortality rate among patients with ACLF^11^. Our study found that DPMAES not only adsorbs bilirubin, albumin-bound toxins, cytokines, but also replenishes essential albumin and coagulation factors, key components for this optimal environment. Compared to the control group, the observation group exhibited a more significant greater decrease in TBiL levels (△*P* < 0.01) and a more stable increase in albumin with significant difference (△*P* = 0.002). The observation group also showed faster clearance of IL-6 (t = 5.046, *P* < 0.0001) and a more pronounced decrease in PCT (t = 4.66, *P* < 0.0001). These findings indicate that DPMAES creates a more favorable internal environment for liver function recovery. Importantly, these effects translate to enhanced liver function. Furthermore, supporting evidence comes from studies conducted by Professor Tong Yan, et al., which demonstrated that the combination of PE with DPMAS or combination of DPMA with PE is more effective in treating ACLF than single treatment modalities^12,13^. The scientific combination therapy of artificial liver is the result of the inheritance and innovation of artificial liver technology. In our subsequent clinical research, complex modes such as coupled plasma filtration adsorption (CPFA), DPMAES, and plasma diafiltration (PDF) have been combined early in the intervention of liver failure, continuing to demonstrate good clinical efficacy. A recent retrospective study found that the short-term effect of sequentially combined multimodal artificial liver treatment (SCMALT) on HBV-ACLF is notable. This combination therapy can more effectively remove inflammatory mediators and lower the model for end-stage liver disease (MELD) score in HBV-ACLF patients, thereby significantly improving the prognosis of these patients, with less effect on the platelet counts^14^.

While the “three-hit” and “second-hit” hypothesis^15,16^ explain aspects of liver failure, cytokine storms play a crucial role in its development^17,18^. IL-6, a key inflammatory cytokine, regulates T-cell activation, differentiation, and hepatocyte regeneration. In inflammatory responses, IL-6 and its receptor IL-6R are essential for recruiting inflammatory cells. Because of the high expression of IL-6 receptors in hepatocytes, IL-6 is the primary cytokine inducing the production of acute-phase proteins^19,20^, including CRP, fibrinogen, hepcidin, haptoglobin, serum amyloid. IL-6 can also exert a protective effect on the liver by promoting hepatocyte regeneration or activating anti-apoptotic pathways, such as inducing Bcl-xL expression to inhibit apoptosis. Jin et al.^21^ found that short-term application of IL-6 can promote hepatocyte regeneration, while prolonged use can make them vulnerable to injury. The study^22^ showed significantly elevated IL-6, IL-10, and IL-17 in liver dysfunction with sepsis, linking them to coagulation issues and sepsis development. Our study suggested that DPMAES uniquely inhibits the inflammatory response through its continuous plasma exchange and dual molecular adsorption, leading to sustained inflammatory factor reduction, improved internal environment, and enhanced liver immune activation. In our study, DPMAES treatment resulted in a significantly greater decrease in IL-6 (t = 5.046, *P* < 0.0001) and a larger reduction in PCT (t = 4.66, *P* < 0.0001) compared to the control group, suggesting its effectiveness in cytokine storm inhibition, organ protection, and improvement of 90-day survival in ACLF patients.

AFP reflects the regenerative capacity of liver cells. In patients with ACLF, particularly young newly diagnosed ones, changes in AFP levels influence both treatment evaluation and prognosis prediction. Lower AFP levels are associated with higher mortality risk^23^. For patients with HBV-ACLF where AFP levels are elevated by less than fivefold, including non-cirrhotic Type A or B liver failure patients, we found that the 60-day survival rate is less than 10%. A recent animal study found that the mechanism of difficult liver regeneration in HBV-ACLF is related to inhibiting the activation of FGFR2mRN and ERK1/2 signaling pathway^24^. Our study showed that DPMAES, compared to PE therapy alone, had a more pronounced protective effect against platelet loss and albumin consumption. Given that patients with liver failure frequently have impaired coagulation functions and thrombocytopenia, prolonged therapy may increase the risk of tubing and filter blockages. Anticoagulation strategy is a critical and debated topic in liver failure treatment. Some scholars have proposed regional citrate anticoagulation as a potential solution, which remains under investigation. Beyond immediate benefits, our study also focused on identifying markers to predict treatment efficacy and potential risks. We monitored key markers like AFP, platelet count, albumin, lactate, and adverse events, as their changes can shed light on overall treatment efficacy and risk in patients. Except in type C ACLF, a gradual decline in both platelet count and albumin levels, along with a slow increase in lactate after treatment, predict poor outcomes and increased risk of hypotension, bleeding, shock, infection, and hepatic encephalopathy during future artificial liver therapy. The dynamic changes in these three markers hold certain significance for guiding early preparation for liver transplantation.

In summary, DPMAES outperforms PE in reducing TBil and albumin-binding toxins, improving coagulation function, suppressing inflammatory storms, and enhancing the internal environment. It significantly improves the 90-day survival rate in ACLF patients. Additionally, DPMAES is superior to the PE treatment in maintaining platelet stability and reducing albumin consumption. Therefore, it is worthy of clinical promotion and application. Future studies with larger samples, multicenter participation, and prospective cohorts are needed to further validate our findings.

## Data Availability

The original contributions presented in the study are included in the article, further inquiries can be directed to the corresponding author.
